# Body surface area as a key determinant of aortic root and arch dimensions in a population-based study

**DOI:** 10.3892/etm.2012.839

**Published:** 2012-11-28

**Authors:** YAN-LI WANG, QING-LING WANG, LIANG WANG, YING-BIAO WU, ZHI-BIN WANG, JAMES CAMERON, YU-LU LIANG

**Affiliations:** 1 Department of Cardiology, Shanghai East Hospital, Tongji University, Shanghai 200120;; 2 Department of Cardiology, Anyang People’s Hospital, Anyang, Henan 455000, P.R. China;; 3 Department of Medicine, Monash Medical Centre, Monash Cardiovascular Research Centre, MonashHeart and Monash University, Clayton, Victoria 3168, Australia

**Keywords:** aortic root, arch dimension, chinese population

## Abstract

The associations between the aortic dimensions (of the aortic sinus, aortic annulus and aortic arch) and physiological variables have not been established in the Chinese population. The present study examined the associations among physiological variables to determine the aortic root and arch dimensions echocardiographically. The diameters of the aortic sinus, annulus and arch were measured in 1,010 subjects via 2-D echocardiography with a 3.5-MHz transducer in a trans-thoracic position. The images of the aortic sinus and aortic annulus were obtained from a standard parasternal long-axis view. The maximum diameter of the valve orifice was measured at the end of systole. The aortic arch dimension was visualized in the long-axis using a suprasternal notch window and the maximum transverse diameter was measured. Epidata 3.0, Excel 2007 and SPSS version 17.0 were used to collect and analyze the data. A total of 1,010 subjects were enrolled. The mean age was 55.0±17.0 years (range of 18 to 90 years). The body surface area (BSA) was the best predictor of all the studied physiological variables and may be used to predict aortic sinus, annulus and arch dimensions independently (r=0.54, 0.37 and 0.39, respectively). Gender, blood pressure, age and BSA are significant predictors of the aortic dimensions. Of these, BSA was the best predictor.

## Introduction

A number of studies on the association between aortic dimensions (of the aortic sinus, aortic annulus and aortic arch) and body physiological variables in Caucasian cohorts have been published. However, whether or not the associations are the same in Chinese populations is unclear. Furthermore, the results of these studies are controversial.

As the outflow track from the left ventricle, the aortic root has an important clinical function. Pathophysiological changes in the aortic root size may result in aortic regurgitation and dissection. Although the aortic size may be accurately determined by 2-D echocardiography, the physiological variables affecting the aortic dimensions which are usable in any indexing procedure remain unclear. A number of studies investigating this association have been published (1–3). However, the subjects have been almost entirely Western, rather than Chinese, individuals. The applicability of the previous results to individuals of Chinese ethnicity remains unclear.

Bella *et al* reported that heredity may explain a substantial proportion of the variability of aortic root size that is not accounted for by age, gender, body size and blood pressure (1). Other studies have identified age, body surface area (BSA), weight and height to be the main determinative factors (2,3). In particular, height has been suggested to be the most important determinant of aortic root size compared with BSA or weight (4–6). Hypertension has also been frequently reported to increase the diameters of large arteries, including the aortic arteries (7–9). However, the effect of blood pressure on large vessel diameter and its correlation with the aortic root dimensions and BSA require further clarification. This correlation is particularly important in older populations since aging is known to increase the stiffness of the aortic wall, as well as the diameter and tortuosity of the aorta itself.

The results of previous studies remain controversial. Furthermore, research investigating the dimensions of the aortic root and physiological factors in a large ethnic group such as the Chinese population are not available. The present study investigated the associations among morphological and physiological variables to determine the aortic root and aortic arch dimensions echocardiographically in a population-based study.

## Materials and methods

### Sample

A total of 1,010 Chinese patients were recruited from the Yancheng First People’s Hospital (Jiangsu, China). The study was conducted in accordance with the Declaration of Helsinki and with approval from the Ethics Committee of Shanghai East Hospital of Tongji University. Written informed consent was obtained from all participants. Patients with cardiovascular diseases, including calcified aortic valves, aortic stenosis and regurgitation, mitral valve dysfunction, left ventricular dysfunction and Marfan syndrome, or other cardiac-related diseases confirmed by echocardiography, were excluded from the study.

### Echocardiographic measurements

**Figure 1.**
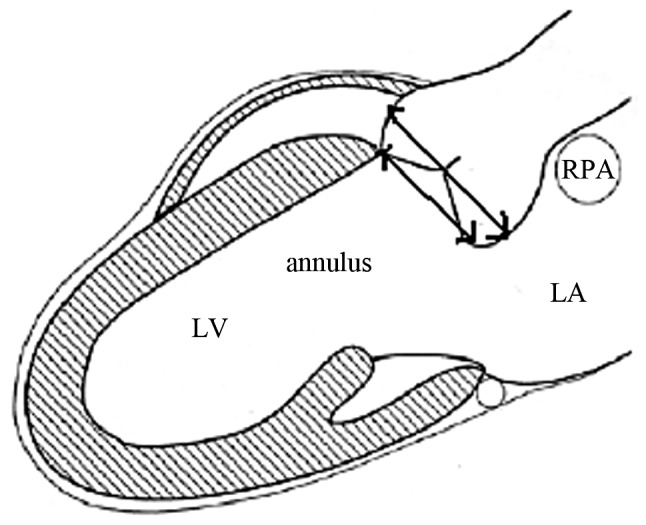
Sites of diameter measurements of the aortic sinus and annulus. The images were obtained from a standard parasternal long-axis view with the maximum diameter of the valve orifice measured at the end of systole with 2-D echocardiography. LA, left atrium; LV, left ventricle; RPA, right pulmonary artery.

**Figure 2.**
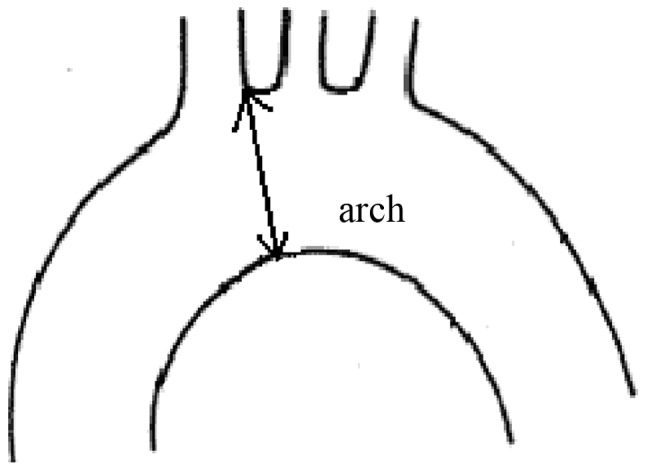
Site of diameter measurements of the aortic arch. Images were obtained from standard and suprasternal notch views by 2-D echocardiography.

All subjects underwent echocardiography using a high-resolution ultrasound machine (Philips Sonos 7500, Hewlett Packard, NY, USA) with a 3.5-MHz transducer in a trans-thoracic position. The images of the aortic sinus and aortic annulus were obtained from a standard parasternal long-axis view with the maximum diameter of the valve orifice measured at the end of systole (Fig. 1). The aortic arch was visualized in the long-axis view using a suprasternal notch window and the maximum transverse diameter was measured as shown in Fig. 2. Each image was measured three times to ensure accuracy. The data were then analyzed using the echocardiograph machine analysis system (PHILIP SSD-7500) and the images were obtained from the same position. The echocardiographic data were recorded and analyzed by a single observer.

### Clinical measurements

Systolic blood pressure (SBP) and diastolic blood pressure (DBP) were recorded at phases I and V of the Korotkoff sounds. The height and weight obtained from the baseline examination were used to calculate the body mass index (BMI) [weight in kg/(height in m)^2^]. BSA was calculated using the Du Bois (10) formula:

$$\text{BSA}=\left[\text{weight}{\left(\text{kg}\right)}^{0.425}\times \text{height}{\left(\text{cm}\right)}^{0.725}\times 71.44\right]/10,000$$

### Statistical analysis

Statistical analysis was performed using Microsoft Excel 2007 and SPSS version 17.0. Continuous variables are expressed as the mean ± SD. Group comparison was performed with the independent t-test, whereas Pearson’s correlation was used to determine the correlations between physiological variables and aortic dimensions. Stepwise multiple linear regression analysis was performed to compare the association between dependent (aortic sinus, aortic arch, aortic annulus) and independent variables. P<0.05 was considered to indicate a statistically significant difference.

## Results

### General data

A total of 1,010 subjects (461 female and 549 male) aged 55.0±17.0 years (range = 18–90 years) were enrolled. The subjects from the two groups were similar with regard to age, SBP and pulse pressure (PP; all P>0.05). Height, weight, BSA, DBP and the diameters of the aortic sinus, annulus and aortic arch were significantly greater in male subjects than in female subjects (P<0.001, Table I).

### Correlation analysis

**Figure 3.**
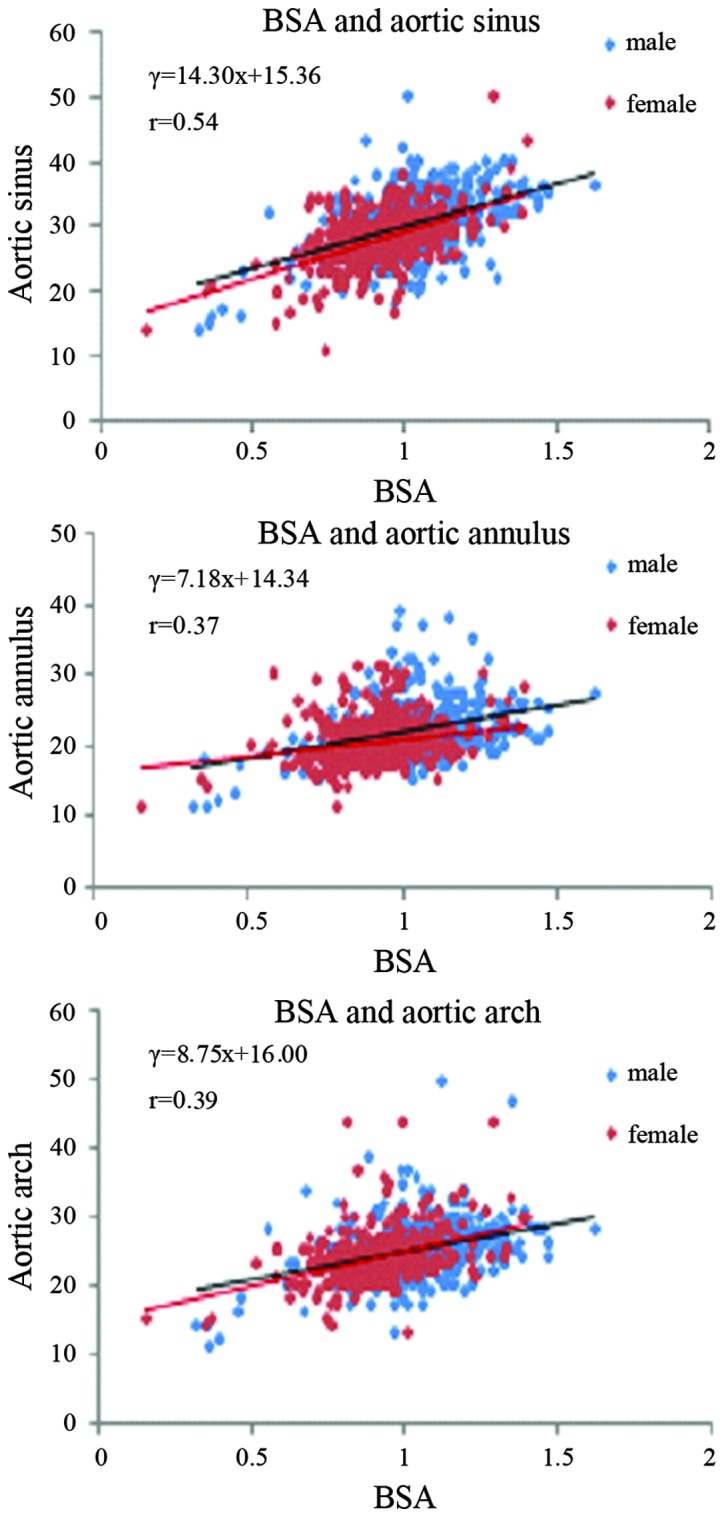
Equation and scatter plots of BSA and aortic diameters for males and females. BSA, body surface area.

The correlation analysis results revealed that the aortic root and arch dimensions were weakly correlated with age, gender, height, weight, BSA, BMI, DBP and SBP (P<0.05). PP did not correlate with the size of the aortic annulus (Table II). Stepwise regression analysis results showed that BSA was the best parameter for independently predicting the aortic sinus, annulus and arch dimensions with the normative diameters at 14.30xBSA+15.36 (r=0.54), 7.18xBSA+14.34 (r=0.37) and 8.75xBSA+16.00 (r=0.39), respectively (Fig. 3).

In addition to BSA (β coefficient = 2.71), the stepwise regression results revealed that BMI and height were independently associated with the size of the aortic sinus. Their *β* coefficients were −1.89 and −1.22, respectively (Table III). This result indicates that BMI and height have negative correlations with the size of the aortic sinus.

## Discussion

The aim of the present study was to assess the dimensions of the proximal aorta, including the aortic root, aortic sinus and aortic annulus, and of the aortic arch in relation to physiological variables in a population-based sample. The present study, which is unique since it involved Chinese subjects, identified a linear correlation between BSA and the maximum diameters of the aortic root (at the sinus and annulus levels) and arch. The correlation coefficients were 0.54, 0.37 and 0.39, respectively (P<0.01). This finding is consistent with the study by Roman *et al*(11), who demonstrated that BSA is a valuable predictor of aortic root dilatation at the aortic sinus in children. The correlation coefficient in their study was 0.93 (P<0.0005). The data of the present study further suggested that BSA has a stronger correlation with the aortic sinus dimension compared with the aortic arch and annulus. This result is in agreement with previous studies, in which logarithmic correlations between BSA and the aortic dimensions at the level of the aortic annulus, sinus, sinotubular junction, before the origin of the innominate artery, before and after the origin of the left carotid artery, and after the left subclavian artery and descending aorta, have all been reported in children and young adults (6,12).

In addition to BSA, gender may be crucial in determining aortic dimensions. Differences in aortic dimensions between males and females have been previously reported (13,14). The aortic dimensions at the aortic sinus and at the level of the sinotubular junction in males were greater than those in females, with the exception of the group with the smallest BSA measurements (0.5–0.75 m^2^) (12). The study by Vasan *et al* also reported that the aortic root diameter in males was greater than in females (8) and an association was observed between the aortic sinus dilatation and male gender (15). These findings are consistent with those of the present study, which showed a significant larger aortic size in males than in females.

The results from the analysis of the present data showed that age was also a predictor of aortic sinus size. This finding was consistent with the results from the study by Vasan *et al*(8), which revealed that the aortic diameter increased by 0.8 mm in males and 0.9 mm in females every ten years. This generalized aortic dilatation may be associated with debris and fragmentation of the vessel wall, resulting in reduced flexibility. However, the effect of age on the aortic dimensions may be more complicated than previously thought, particularly in older populations since height significantly decreases during the aging process. A marked determinant of the aortic dimensions or the true effect of age on the actual aortic dimensions in older populations has yet to be clarified as height is closely associated with BSA.

Blood pressure is another variable that may affect aortic diameter. Although the findings of the present study are significant, the data revealed only a weak association between blood pressure and the aortic dimensions (aortic root and arch). Biaggi *et al*(14) hypothesized that systemic hypertension may alter the dimensions of the ascending aorta and aortic sinus. Other researchers have suggested that blood pressure has no independent effect on aortic size at the level of the annulus, sinus, supra-aortic ridge and proximal ascending aorta (11). Palmieri *et al*(15) reported that DBP was weakly but independently correlated with the aortic sinus diameter but not the categorical variable, indicating that the aortic sinus was dilated. The correlation of blood pressure with the sinus diameter may be slightly more marked than that identified by the authors since the sinus diameter and DBP affect each other (13). Kim *et al*(16) observed that hypertension was associated with a significant but small increase in the the aortic root size. Although aortic diameters were most closely associated with BSA in an age-matched population, the diameters increased with increasing levels of blood pressure.

The present study had limitations. For example, smoking and diabetes mellitus were not taken into consideration. Palmieri *et al*(15) reported that a history of smoking was not associated with aortic sinus dilatation. However, Chen *et al*(17) showed that patients with diabetes had significantly smaller aortic root dimensions compared with patients without diabetes.

Another limitation of the present study was that physiological parameters such as ejection fraction and ventricular mass were not included and thus any associations between cardiac function and the aortic root or arch were not evaluated. This association may be important since Palmieri *et al*(15) reported sinus dilatation was associated with higher left ventricular mass and lower systolic function, both of which may contribute to higher cardiovascular risk in subjects with aortic root dilatation.

Other methods may be used to measure the aortic root dimensions, including computed tomography and magnetic resonance imaging. However, these are limited in practicability due to factors such as radiation exposure and high cost. However, ultrasound is readily available and popular due to its relatively low cost. As early as 1975, Francis *et al*(13) considered that the associativity of echocardiographic and practical data was 0.70 and el Habbal *et al*(18) reached a similar conclusion, suggesting that the 2-D imaging results almost completely matched with the data obtained via surgical measurement.

Finally, all images in the present study were measured via 2-D echocardiography, whereas other studies used M-mode echocardiography. Although 2-D images produce larger aortic sinus measurements than those of M-mode images (P<0.05 in all BSA groups), the measurements made by the two methods correlated well (r=0.87, P<0.001) (12) which may explain the small differences in aortic measurements between the present study and previous studies.

The present study investigated the aortic dimensions in relation to physiological variables in a population-based study. Of all the variables studied, BSA was identified to be the best predictor of the aortic dimensions.

## Figures and Tables

**Table I.** Baseline characteristics of the study population.

Variable	Age (years)	Height (cm)	Weight (kg)	BMI (kg/m^2^)	BSA	SBP (mmHg)	DBP (mmHg)	PP (mmHg)	D-sinus (mm)	D-annulus (mm)	D-arch (mm)
Total (1010)	55.0±17.0	164.±10.2	131.±24.9	24.1±3.7	1.0±0.17	133.±24.9	83.8±13.4	47.0±19.8	29.5±4.5	21.4±3.3	24.6±3.8
Male (549)	55.4±18.1	169.3±9.8	139.9±24.3	24.3±3.8	1.0±0.16	134.5±21.3	85.3±13.3	49.2±15.3	30.6±4.6	22.1±3.3	25.1±3.8
Female (461)	54.4±15.7	159.2±7.6	121.3±21.7	23.8±3.6	0.9±0.15	132.1±23.9	81.9±13.4	50.3±18.1	28.1±4.1	20.5±3.1	24.1±3.6
P-value	0.370	<0.001	<0.001	0.030	<0.001	0.097	<0.001	0.289	<0.001	<0.001	<0.001

BMI, body mass index; BSA, body surface area; SBP, systolic blood pressure; DBP, diastolic blood pressure; PP, pulse pressure; D -sinus, D-sinus-dimension of aortic sinus; D -annulus, D-annulus-dimension of aortic annulus; D -arch, D-arch-dimension of aortic arch.

**Table II.** Correlation coefficients (r) between the dimension of the aortic root, aortic arch and physiological factors.

Dimension	Age (years)	Height (cm)	Weight (kg)	BMI (kg/m^2^)	BSA	SBP (mmHg)	DBP (mmHg)	PP (mmHg)	Gender
Sinus	0.32^a^	0.41^a^	0.41^a^	0.23^a^	0.54^a^	0.23^a^	0.24^a^	0.12^a^	0.28^a^
Annulus	0.23^a^	0.35^a^	0.34^a^	0.16^a^	0.37^a^	0.08^b^	0.09^a^	0.04	0.24^a^
Arch	0.32^a^	0.30^a^	0.34^a^	0.20^a^	0.39^a^	0.17^a^	0.13^a^	0.15^a^	0.13^a^

a P≤0.01,

b 0.01<P<0.05. BMI, body mass index; BSA, body surface area; SBP, systolic blood pressure; DBP, diastolic blood pressure; PP, pulse pressure.

**Table III.** Stepwise multiple linear regression (aortic sinus).

Lead-in Variables	R	R^2^	P-value	β coefficient
BSA	0.52	0.27	P<0.01	0.52
BSA, BMI	0.60	0.37	P<0.01	0.90,−0.49
BSA, BMI, height	0.75	0.56	P<0.01	2.82,−1.94,−1.26
BSA, BMI, height, age	0.77	0.59	P<0.01	2.75,−1.90,−1.23,0.16
BSA, BMI, height, age, DBP	0.78	0.59	P<0.01	2.71,−1.89,−1,22,0.15,0.07

BMI, body mass index; BSA, body surface area; DBP, diastolic blood pressure.

## References

[1] Bella JN, MacCluer JW, Roman MJ (2002). Genetic influences on aortic root size in American Indians: the Strong Heart Study. Arterioscler Thromb Vasc Biol.

[2] Tamás E, Nylander E (2007). Echocardiographic description of the anatomic relations within the normal aortic root. J Heart Valve Dis.

[3] Zhu D, Zhao Q (2008). Aortic valve annulus and sinus-tube joint diameters in normal adults of Chinese Han ethnic group. Chin Med J (Engl).

[4] Meuleman C, Boccara F, Nguyen XL (2008). Is the aortic root dilated in obstructive sleep apnoea syndrome?. Arch Cardiovasc Dis.

[5] Reed CM, Richey PA, Pulliam DA, Somes GW, Alpert BS (1993). Aortic dimensions in tall men and women. Am J Cardiol.

[6] Nidorf SM, Picard MH, Triulzi MO (1992). New perspectives in the assessment of cardiac chamber dimensions during development and adulthood. J Am Coll Cardiol.

[7] O’Rourke MF, Nichols WW (2005). Aortic diameter, aortic stiffness, and wave reflection increase with age and isolated systolic hypertension. Hypertension.

[8] Vasan RS, Larson MG, Levy D (1995). Determinants of echocardiographic aortic root size. The Framingham Heart Study. Circulation.

[9] Jondeau G, Boutouyrie P, Lacolley P (1999). Central pulse pressure is a major determinant of ascending aorta dilation in Marfan syndrome. Circulation.

[10] Du Bois D, Du Bois EF (1989). A formula to estimate the approximate surface area if height and weight be known. 1916. Nutrition.

[11] Roman MJ, Devereux RB, Kramer-Fox R, O’Loughlin J (1989). Two-dimensional echocardiographic aortic root dimensions in normal children and adults. Am J Cardiol.

[12] Poutanen T, Tikanoja T, Sairanen H, Jokinen E (2003). Normal aortic dimensions and flow in 168 children and young adults. Clin Physiol Funct Imaging.

[13] Francis GS, Hagan AD, Oury J, O’Rourke RA (1975). Accuracy of echocardiography for assessing aortic root diameter. Br Heart J.

[14] Biaggi P, Matthews F, Braun J, Rousson V, Kaufmann PA, Jenni R (2009). Gender, age, and body surface area are the major determinants of ascending aorta dimensions in subjects with apparently normal echocardiograms. J Am Soc Echocardiogr.

[15] Palmieri V, Bella JN, Arnett DK (2001). Aortic root dilatation at sinuses of valsalva and aortic regurgitation in hypertensive and normotensive subjects: The Hypertension Genetic Epidemiology Network Study. Hypertension.

[16] Kim M, Roman MJ, Cavallini MC, Schwartz JE, Pickering TG, Devereux RB (1996). Effect of hypertension on aortic root size and prevalence of aortic regurgitation. Hypertension.

[17] Chen XF, Wang JA, Lin XF (2009). Diabetes mellitus: is it protective against aortic root dilatation?. Cardiology.

[18] el Habbal M, Somerville J (1989). Size of the normal aortic root in normal subjects and in those with left ventricular outflow obstruction. Am J Cardiol.

